# MicroRNA-22 (miR-22) Regulates Trophoblast Cell Invasion via the Specificity Protein 1 (Sp1)/Cystathionine β-Synthase (CBS)/Matrix Metalloproteinases 2 and 9 (MMP-2 and MMP-9) Pathway: Implications for the Pathogenesis of Preeclampsia

**DOI:** 10.7759/cureus.99884

**Published:** 2025-12-22

**Authors:** Pallavi Arora, Sahil Kalra, Renu Dhingra, Pallavi Kshetrapal, Neerja Bhatla, Sadanand Dwivedi

**Affiliations:** 1 Department of Anatomy, Shri Mata Vaishno Devi Institute of Medical Excellence, Katra, IND; 2 Department of Anatomy, All India Institute of Medical Sciences, New Delhi, New Delhi, IND; 3 Department of Mechanical Engineering, Indian Institute of Technology Jammu, Jammu, IND; 4 Department of Maternal and Child Health, Translational Health Science and Technology Institute, Faridabad, IND; 5 Department of Obstetrics and Gynaecology, All India Institute of Medical Sciences, New Delhi, New Delhi, IND; 6 Department of Biostatistics, All India Institute of Medical Sciences, New Delhi, New Delhi, IND

**Keywords:** cystathionine β-synthase (cbs), matrix metalloproteinase 2, matrix metalloproteinase 9, micro-rna 22, preeclampsia (pe), specificity protein 1 (sp1)

## Abstract

Altered matrix metalloproteinases (MMPs) 2 and 9 have been documented in pregnancy complications, notably in conditions such as preeclampsia (PE), particularly early-onset preeclampsia (EOPE). The MMPs play a crucial role in regulating the invasion of trophoblast cells. In the present study, we delved into the upstream mechanisms involving microRNA (miRNA) and their targets that influence the synthesis of hydrogen sulfide enzymes (cystathionine β-synthase; CBS) and MMPs, subsequently impacting trophoblast cell invasion. Functional cellular assays, involving both gain and loss of function, were performed on HTR-8/SVneo cells, focusing on the expression of microRNA-22-3p (miR-22-3p), specificity protein 1 (Sp1), cystathionine β-Synthase (CBS), MMP-2, and MMP-9. The invasive capacity of the cells was evaluated using a transwell invasion assay, with levels of mRNA and protein determined by Real-time quantitative reverse transcription polymerase chain reaction (qRT-PCR), immunoblot, and immunofluorescence. Transfecting cells with the miR-22-3p mimic significantly decreased the mRNA and protein expression of Sp1, CBS, MMP-2, and MMP-9. Conversely, transfection with a Sp1 overexpression construct led to a notable upregulation of CBS, MMP-2, and MMP-9 expression. Furthermore, exposing cells to an exogenous H₂S donor (sodium hydrogen sulfide (NaHS)) significantly increased MMP-2 and MMP-9 levels. To our knowledge, this study represents the first evidence demonstrating that the miR-22/Sp1/CBS/MMPs 2 and 9 axis regulates trophoblast cell invasion.

## Introduction

MicroRNAs (miRNAs) are small endogenous, single-stranded RNAs that post-transcriptionally regulate the expression of various target genes [1]. Global studies indicate the significance of miRNAs as crucial regulators of placental development [2-8], and their presence has been identified in human placental tissues [9]. Previous research demonstrates that miRNAs regulate various aspects of trophoblast cell behavior, including proliferation, apoptosis, invasion, migration, and angiogenesis [4,7,8,10,11]. Abnormal miRNA expression in the placenta, in compromised pregnancies such as preeclampsia (PE), has been reported [3,4,12,13]. Specifically, miR-22 expression was significantly upregulated in the placentae from pregnancies complicated by early-onset preeclampsia (EOPE), compared to control placentae from preterm labor [14]. Specificity protein 1 (Sp1) expression is regulated by miR-22 and has been recognized as a direct target of Sp1 [15], and an inverse linear correlation between miR-22 and Sp1 mRNA expression has been observed in gastric tumors. Also, miR-22 has been shown to decrease CD147 (cluster of differentiation 147) expression by targeting Sp1, contributing to its tumor-suppressor-like activity [16]. Studies in Drosophila SL2 cells have indicated that both human cystathionine β-synthase (CBS) gene promoters (-1a and -1b) are transactivated by Sp1 and Sp3, with a site-specific, synergistic regulatory interaction observed only in the -1b promoter [17]. 

CBS is among the three endogenous enzymes responsible for producing H₂S, through one-carbon unit metabolism and the transfer-sulfur pathway, utilizing the essential amino acid cysteine in vivo [18-21].

H₂S is the third gas signaling molecule in the human body, exerting powerful effects on endothelial cells, smooth muscle cells, inflammatory cells, and cell organelles. Like nitric oxide (NO), H₂S has emerged as a critical cardiovascular signaling molecule that profoundly impacts cardiovascular homeostasis and health [22]. Exogenous H₂S has been reported to promote cell proliferation and invasion by upregulating the expression of matrix metalloproteinases (MMPs) 2 and 9 in human bladder cancer EJ cells [23]. The invasion cascade exhibited by placental trophoblasts has many similarities with cancerous cells, involving extracellular matrix degradation mediated by MMPs [24]. Notably, MMP-2 and MMP-9 play a crucial role in regulating trophoblast invasion [25], and their expression is found to be downregulated in preeclamptic and IUGR placentae [26].

In this study, we investigated the upstream pathways, paying particular attention to microRNA and its targets. Our research concentrated on their function in controlling the H₂S-synthesizing enzyme (notably CBS) and MMPs, which may have consequences for trophoblast cell invasion.

This article was previously posted to the bioRxiv preprint server on March 14, 2023 (doi: https://doi.org/10.1101/2023.03.08.531738).

## Materials and methods

Cell culture

The human first-trimester trophoblast cell line (HTR-8/SVneo) was procured from the American Type Culture Collection at the fifth passage and maintained in Dulbecco's Modified Eagle Medium and Ham’s F12 medium in a 1:1 ratio, supplemented with 10% fetal bovine serum, 100 U/mL penicillin, 100 µg/mL streptomycin, and 250 µg/mL amphotericin B at 37°C with 5% CO₂ in a tri-gas CO₂ incubator (Thermo Fisher Scientific, Waltham, MA, USA). Cells were passaged using 0.25% trypsin and 0.01% ethylenediaminetetraacetic acid (EDTA).

Cell transfection and treatments

HTR-8/SVneo cells were cultured in six-well plates and grown to 80% confluency. Cells were transfected with miR-22-3p mimic (hsa-miR-22-3p mirVana miRNA mimic, Ambion, Austin, TX, USA), miR-22-3p inhibitor (MISSION Synthetic miRNA Inhibitor, Sigma Aldrich, Burlington, MA, USA), and Sp1 overexpression construct (Thermo Fisher Scientific) using Lipofectamine (RNAiMAX transfection reagent (Thermo Fisher Scientific)) in Opti-MEM (Gibco, Waltham, MA, USA). Cells were treated with Sp1 inhibitor (2,4,5-trifluoroaniline, Sigma Aldrich), H₂S donor (sodium hydrogen sulfide (NaHS), Cayman Chemical Company, Ann Arbor, MI, USA), and CBS inhibitor (aminooxyacetic acid (AOAA), Sigma Aldrich). Optimal doses were standardized for the respective experiments (Tables 1-2).

**Table 1:** Cell transfection: standardized doses for one reaction.

	Nucleic acid	Lipofectamine (RNA iMAX transfection reagent)	Opti-MEM
hsa-miR-22-3p mirVana miRNA mimic	12.5 µL (198.5 ng/µL)	3 µL	150 µL
MISSION Synthetic miRNA Inhibitor (Sigma)	10 µL (238.9 ng/µL)	3 µL	150 µL
Specificity protein 1 (Sp1) Plasmid (Thermo)	2.54 µL (954.9 ng/µL)	3 µL	150 µL

**Table 2:** Cell treatments: standardized doses. CBS, cystathionine β-synthase; AOAA, aminooxyacetic acid; NaHS, sodium hydrogen sulfide

	Concentration	Duration
Sp1 inhibitor (2,4,5 Trifluoroaniline)	250 µM	5 minutes
CBS mimic/H_2_S donor (NaHS)	100 µM	5 minutes
CBS inhibitor (AOAA)	50 µM	5 minutes

Transwell invasion assay 

The invasive capacity of cells was assessed by the transwell invasion assay. To prepare the transwell inserts, 100 µL of diluted Matrigel (Corning, Corning, NY, USA) was added to the insert (Greiner Bio-One, Frickenhausen, Germany) and incubated at 37°C overnight. Then, 750 µL of culture medium (DMEM, Ham’s F-12 medium with 10% FBS) was added to the lower chamber. Approximately 2.5 × 10⁴ cells (after various transfections and treatments) in serum-free medium (DMEM and Ham’s F-12 medium, Gibco) were placed on a transwell insert (pore size 8.0 µm) and incubated at 37°C for 16 hours. Subsequently, the medium was removed, followed by washing with 1× PBS (phosphate-buffered saline), and the cells were fixed with formaldehyde (3.7% in PBS). Permeabilization was performed with 100% methanol for 20 minutes, followed by washing with 1× PBS. Staining was done using crystal violet for 15 minutes at room temperature. Subsequently, the cells were washed with 1× PBS, non-invasive cells were scraped off with cotton swabs, and invasive cells were counted under a Nikon Eclipse Ti-S microscope (Nikon, Tokyo, Japan) using a DS-Fi2 camera. The number of invaded cells was counted on each transwell insert under the 20× objective, and 16 fields were analyzed for each insert.

Calculation of percentage invasion and invasion index

The formulae to calculate percentage invasion are as follows: $\text{Percentage invasion} = \frac{\text{Cells invaded on the lower surface of insert}}{\text{Total number of cells}} \times 100$, and the formula for calculation of the invasion index is: $\text{Invasion Index} = \frac{\text{Number of invaded cells in the test group}}{\text{Number of invaded cells in the control group}} \times 100$.

After miR-22-3p mimic and inhibitor transfection, levels of miR-22-3p were observed by Real-time quantitative reverse transcription polymerase chain reaction (qRT-PCR). mRNA and protein expression of Sp1, CBS, and MMPs 2 and 9 was determined. After Sp1 overexpression construct transfection and its inhibitor treatment, Sp1, CBS, and MMPs 2 and 9 expression was observed at both transcriptional and translational levels. When the cells were subjected to NaHS and AOAA treatments, mRNA and protein expression of CBS and MMPs 2 and 9 was observed.

Real-time quantitative reverse transcription polymerase chain reaction (qRT-PCR)

RNA isolation from cells was done using the miRNeasy Mini Kit, (Qiagen, Hilden, Germany; for gene expression of miR-22-3p), and TRIzol™ Reagent (Invitrogen, Thermo Fisher Scientific; to assess mRNA expression of Sp1, CBS, and MMPs 2 and 9). The quality of RNA was examined by denaturing gel, and quantification was done on a Micro-Volume UV/Visible Spectrophotometer (Thermo Fisher Scientific; NanoDrop™ 2000). cDNA was synthesized using the miScript II RT Kit (Qiagen; for gene expression of miR-22-3p), and the RevertAid H-minus Reverse Transcriptase Kit, (Thermo Fisher Scientific; for mRNA expression of Sp1, CBS, and MMPs 2 and 9). cDNA was subsequently used for qRT-PCR (CFX96 Touch™ Real-Time PCR Detection System; Bio-Rad, Hercules, CA, USA). The miScript SYBR Green PCR Kit (Qiagen) was used with the miScript Primer Assay (Qiagen) to determine the gene expression of miR-22-3p in HTR-8/SVneo cells, with U6 small nuclear RNA as an internal control. qRT-PCR reactions were carried out in a 20 µL volume, including cDNA (template), SYBR Green (Thermo Fisher Scientific), forward and reverse primers (Sigma Aldrich), and nuclease-free water, to amplify cDNA for determining the mRNA expression of Sp1, CBS, and MMPs 2 and 9 in HTR-8/SVneo cells. Glyceraldehyde-3-phosphate dehydrogenase (GAPDH) mRNA was used as an internal control. Primers were designed using the National Center for Biotechnology Information (NCBI) database and confirmed with in-silico PCR (Table 3).

**Table 3:** Primers were designed by NCBI and confirmed with in-silico PCR. Sp1: specificity protein 1; miR-22-3p: microRNA-22-3p; CBS: cystathionine β-synthase; MMP2: matrix metalloproteinase-2; MMP9: matrix metalloproteinase-9; U6: U6 small nuclear RNA; GAPDH: glyceraldehyde-3-phosphate dehydrogenase; NCBI: National Centre for Biotechnology Information; PCR: polymerase chain reaction

Targets	Primers
miR-22-3p	5’-AAGCUGCCAGUUGAAGAACUGU-3’
Sp1	FP - GGAGAGCAAAACCAGCAGAC RP - AAGGTGATTGTTTGGGCTTG
CBS	FP - CTGAAGAACGAAATCCCCAA RP - GCCTCCTCATCGTTGCTCTT
MMP2	FP - CGTCTGTCCCAGGATGACATC RP - ATGTCAGGAGAGGCCCCATA
MMP9	FP - CGCCAGTCCACCCTTGT RP - CAGCTGCCTGTCGGTGAGA
U6	FP - CTCGCTTCGGCAGCACA RP - AACGCTTCACGAATTTGCGT
GAPDH	FP - AGCCGAGCCACATC RP - TGAGGCTGTTGTCATACTTCTC

Immunoblot

Protein was extracted in radioimmunoprecipitation assay (RIPA) buffer (Thermo Fisher Scientific) by adding a protease inhibitor cocktail. Separating and stacking gels were prepared. A 3× non-reducing sample buffer was added to each isolated protein sample from cells (after various transfections and treatments), followed by denaturation at 95°C for five minutes. The samples were loaded into the wells with a protein molecular marker (Thermo Fisher Scientific). Subsequently, the gel was run at 50 V (vertical electrophoresis apparatus, Bio-Rad) in an electrophoresis buffer for 3-3.5 hours until good band separation was achieved. The nitrocellulose membrane was used to transfer gel products onto the membrane in a transfer buffer for 90 minutes. The membrane was washed with TBS-T20 and blocked in 5% bovine serum albumin (BSA) in tris-buffered saline-tween-20 (TBS-T20) for 90 minutes. Overnight incubation was done with primary antibodies: Sp1 (Merck, Darmstadt, Germany) at a dilution of 1:400, CBS (Abcam, Cambridge, UK) at a dilution of 1:1000, MMP-2 (Abcam) at a dilution of 1:1000, and MMP-9 (Abcam) at a dilution of 1:1000, at 4°C. Washing was done with TBS-T20, followed by incubation with secondary antibodies (Abcam) for three hours, and then washed again with TBS-T20. An enhanced chemiluminescence (ECL) kit (Thermo Fisher Scientific) was used to visualize bands on the densitometer (ProteinSimple, San Jose, CA, USA).

Immunofluorescence 

Coverslips were coated with poly-L-lysine for one hour at room temperature. Coverslips were rinsed, dried, and sterilized under UV light. Cells were grown on coverslips and subjected to various transfections and treatments. Cells were briefly rinsed in PBS, and then incubated in 100% methanol (chilled at -20°C) at room temperature for five minutes. Cells were subsequently washed with ice-cold PBS. Antigen retrieval was done at 95°C for 10 minutes, followed by washing in PBS for five minutes. Cells were incubated for 10 minutes with PBS containing 0.1% Triton X-100, then washed in PBS for five minutes. Subsequently, cells were incubated in 1% BSA in PBST (PBS + 0.1% Tween 20) for 30 minutes to block nonspecific binding of the antibodies. Cells were incubated in primary antibodies (Sp1, Merck, 1:50; CBS, Abcam, 1:200; MMP-2, Abcam, 1:100; and MMP-9, Abcam, 1:100) diluted in 1% BSA in PBST overnight at 4°C, followed by three washes with PBS. Subsequently, cells were incubated with secondary antibodies (Sp1, CBS, MMP-2, MMP-9, fluorescein isothiocyanate (FITC)-conjugated, Abcam) diluted in 1% BSA in PBST for one hour at room temperature in the dark, followed by three washes with PBS. The Vector TrueVIEW™ Autofluorescence Quenching Kit (Vector Laboratories, Burlingame, CA, USA) was used to diminish unwanted autofluorescence from non-lipofuscin sources. Mounting was done with Fluoroshield mounting media containing DAPI (Abcam). Stained slides were observed under a Nikon Eclipse Ti-S elements microscope using NiS-AR software (version 5.1).

Statistics

Data were analyzed using STATA 14 (StataCorp LLC, College Station, TX, USA) and GraphPad Prism 8 (GraphPad Software, San Diego, CA, USA). Relative quantification cycles of the gene of interest (ΔCq) were calculated as $\Delta C_q = C_q(\mathrm{target}) - C_q(\mathrm{reference})$. Relative mRNA expression with respect to the internal control gene was calculated using 2^-ΔCq^. Paired t and Wilcoxon matched-pairs signed-rank tests were used to compare the average level of the variable between two groups, and for more than two groups, one-way analysis of variance (ANOVA) with Bonferroni’s post hoc test and Kruskal-Wallis with Dunn’s test were applied. A p-value < 0.05 was considered statistically significant.

## Results

**Figure 1:**
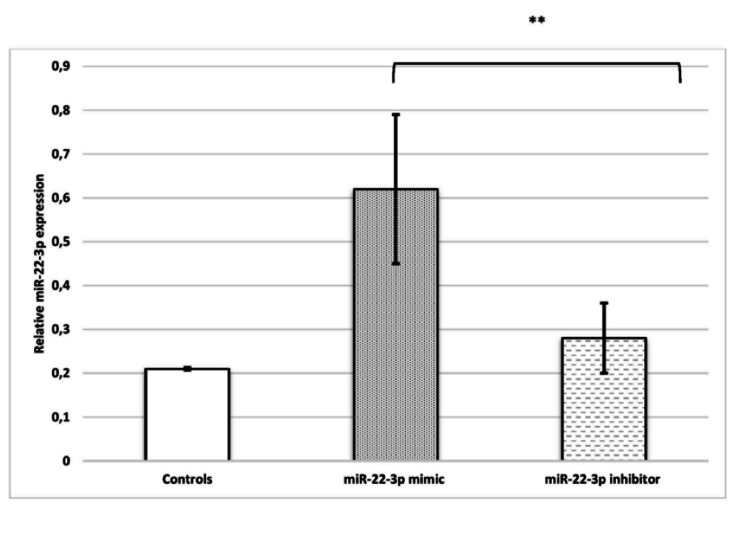
Bar diagrams represent gene expression of miR-22-3p, when no treatment was given (controls), and post-miR-22-3p mimic and inhibitor transfection in HTR-8/SVneo cells. Experiments were performed three times in triplicate (n = 9). Data are presented as mean ± SEM. The Wilcoxon matched-pairs signed-rank test was applied. p ≤ 0.05 was considered statistically significant. ** denotes p ≤ 0.01. miR-22-3p: microRNA-22-3p

miR-22-3p suppresses the invasion of HTR-8/SVneo cells. The role of miR-22-3p in the pathogenesis of PE was studied using mimics and inhibitors. The miR-22-3p mimic induced a noticeable upregulation of miR-22-3p levels. Conversely, miR-22-3p levels were markedly reduced in HTR-8/SVneo cells following transfection with the miR-22-3p inhibitor, indicating that these cell culture systems could be used for gain- and loss-of-function experiments (Figure 1).

**Figure 2:**
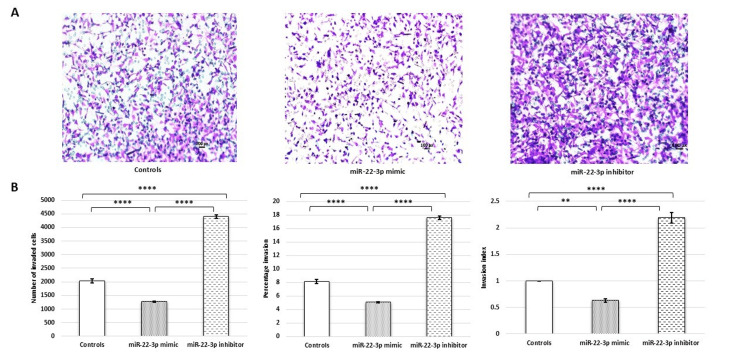
miR 22-3p suppresses invasion of HTR-8/SVneo cells. (A) Representative Matrigel transwell invasion assay images showing invaded cells on the underside of the membrane in controls and after transfection with miR-22-3p mimic and miR-22-3p inhibitor; scale bar: 50 µm. (B) Bar diagrams represent the number of invaded cells, percentage invasion, and invasion index among various experimental groups. Experiments were performed three times in triplicate (n = 9). Data are presented as mean ± SEM. One-way ANOVA, with Bonferroni’s post hoc test, was applied, and p ≤ 0.05 was considered statistically significant. ** denotes p ≤ 0.01; **** denotes p ≤ 0.0001. ANOVA: analysis of variance; miR-22-3p: microRNA-22-3p

Transwell invasion assay analysis revealed a significant reduction in invasion capacity (as indicated by a decrease in the number of invaded cells, percentage invasion, and invasion index) of HTR-8/SVneo cells following the upregulation of miR-22-3p levels, which was partially reversed upon downregulation of miR-22-3p levels (Figure 2).

miR-22-3p targets Sp1, reducing the expression of H₂S-synthesizing enzyme and MMPs. The target genes of miR-22-3p were predicted using public databases (TargetScan, miRanda, miRBase). miR-22-3p binding site sequences were identified in the 3’ UTR of the Sp1 transcript using these tools. In-silico analysis revealed that the miR-22-3p seed region targeted specific areas of the Sp1 3’ UTR.

To understand the function of miR-22-3p and its downstream effectors in the trophoblast cells, gain and loss of function experiments were performed using in-house established assays. Consistent with the in-silico findings, the reduced mRNA and protein expression of Sp1 was observed when the HTR-8/SVneo cells were transfected with miR-22-3p mimic. Contrary to these findings, the mRNA and protein expression of Sp1 were significantly elevated upon treatment with miR-22-3p inhibitor.

**Figure 3:**
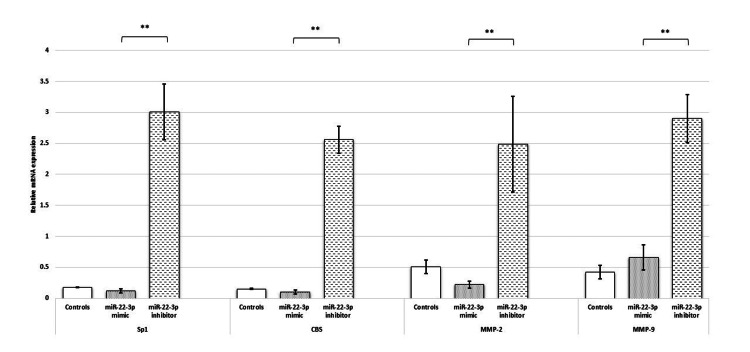
miR-22-3p reduces expression of Sp1, H2S synthesizing enzyme and MMPs in HTR-8/SVneo cells. Bar diagrams represent gene expression of Sp1, CBS, and MMPs 2 and 9 post-miR-22-3p mimic and inhibitor transfection. Experiments were performed three times in triplicate (n = 9). Data are presented as mean ± SEM. The Wilcoxon matched-pairs signed-rank test was applied, and p ≤ 0.05 was considered statistically significant. ** denotes p ≤ 0.01. Sp1: specificity protein 1; CBS: cystathionine β-synthase; H2S: hydrogen sulfide; miR-22-3p: microRNA-22-3p; MMPs: matrix metalloproteinases

Downstream targets of Sp1 were assayed at the transcript level in the placental cell line. A significant reduction in the mRNA expression of CBS, MMP-2, and MMP-9 was observed in HTR-8/SVneo cells transfected with the miR-22-3p mimic. Corroborating these results, significantly elevated mRNA expression of CBS, MMP-2, and MMP-9 was observed when the cells were transfected with the miR-22-3p inhibitor (Figure 3).

**Figure 4:**
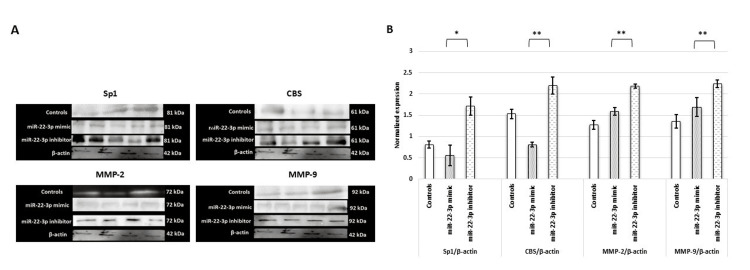
HTR-8/SVneo cells transfected with miR-22-3p mimic show reduced protein expression of Sp1, H2S-producing enzyme (CBS), and MMPs. (A) Representative images of an immunoblot showing the protein expression of Sp1, CBS, MMP-2, and MMP-9 when HTR-8/SVneo cells were transfected with miR-22-3p mimic and inhibitor. (B) Bar diagrams represent the normalized values of Sp1, CBS, MMP-2, and MMP-9 with respect to β-actin (loading control) post-miR-22-3p mimic and inhibitor transfection. Experiments were performed three times in triplicate (n = 9). Data are presented as mean ± SEM. Statistical analysis was done using the Wilcoxon matched-pairs signed-rank test (Sp1) and paired t-test (CBS, MMPs 2 and 9), and p ≤ 0.05 was considered statistically significant. * denotes p ≤ 0.05; ** denotes p ≤ 0.01. Sp1: specificity protein 1; CBS: cystathionine β-synthase; H2S: hydrogen sulfide; miR-22-3p: microRNA-22-3p; MMPs: matrix metalloproteinases

**Figure 5:**
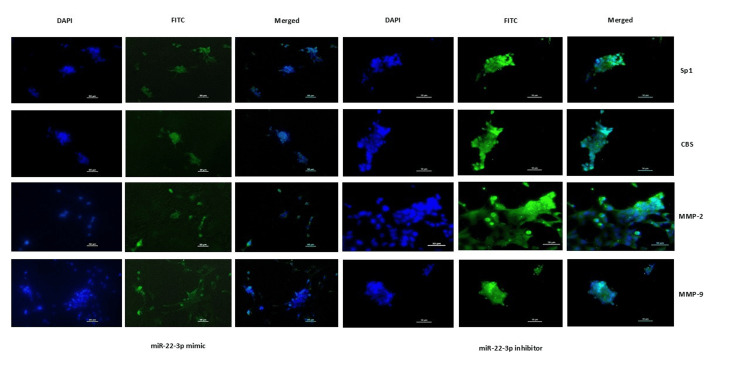
Reduced protein expression of Sp1, H2S-producing enzyme (CBS), and MMPs was observed in HTR-8/SVneo cells transfected with miR-22-3p mimic. Representative immunofluorescence images showing localization of Sp1, CBS, MMP-2, and MMP-9 proteins stained with FITC, when HTR-8/SVneo cells were transfected with miR-22-3p mimic (three left panels) and inhibitor (three right panels). Nuclei were stained with DAPI; scale bar: 50 µm. Sp1: specificity protein 1; CBS: cystathionine β-synthase; H2S: hydrogen sulfide; miR-22-3p: microRNA-22-3p; MMPs: matrix metalloproteinases; FITC: fluorescein isothiocyanate; DAPI: 4′,6-diamidino-2-phenylindole

The transcript data of these target genes (Sp1, CBS, MMP-2, and MMP-9) were validated at the protein level. A significant reduction in the protein expression of all the genes was observed in placental cells transfected with the miR-22-3p mimic, using both immunoblots (Figure 4) and immunofluorescence assays (Figure 5).

**Figure 6:**
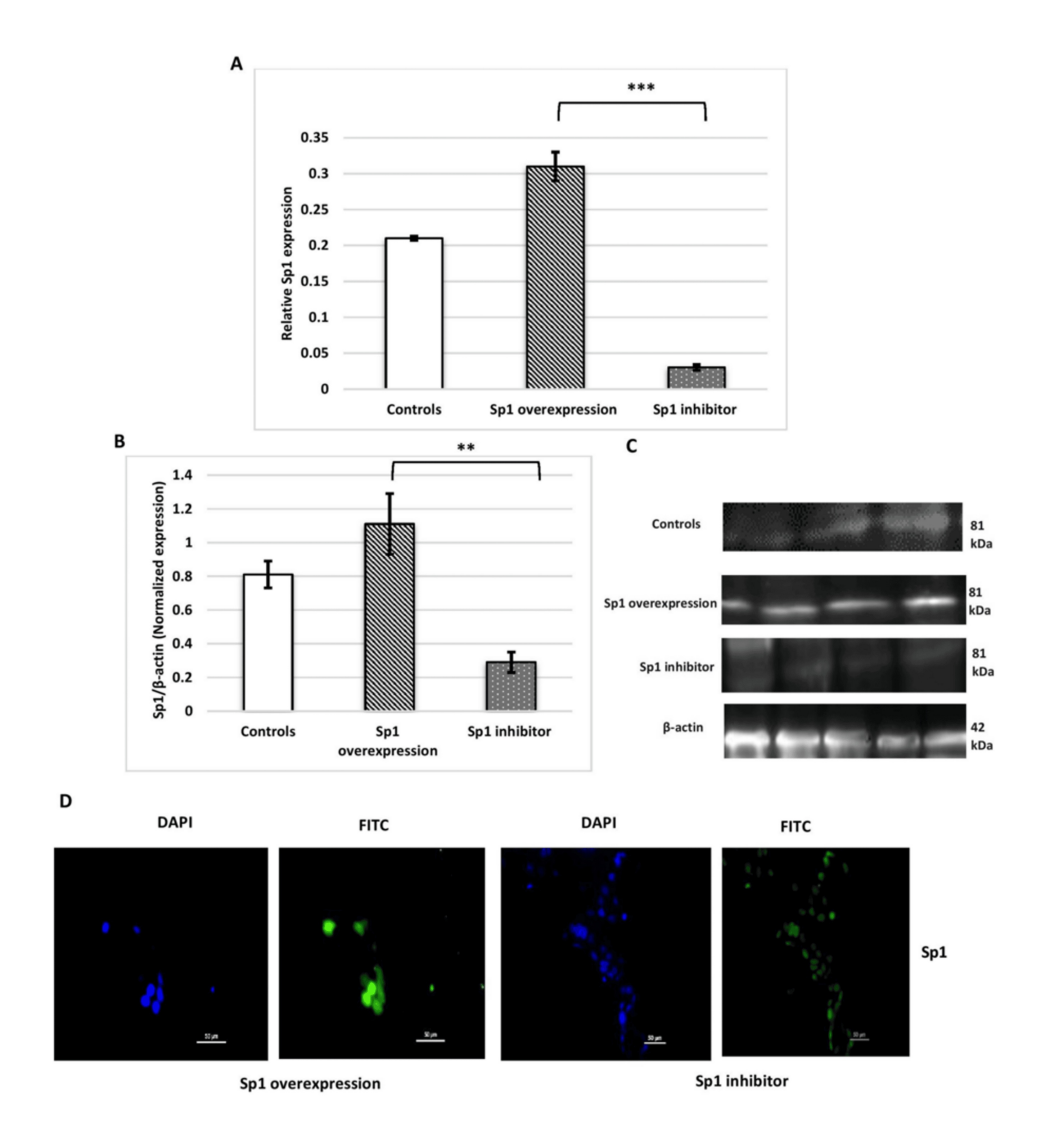
(A, B) Bar diagrams, (C) representative images, and (D) immunofluorescence Bar diagrams represent mRNA (A) and protein expression (B) of Sp1 when no treatment was given (controls), post-Sp1 mimic transfection, and treatment with Sp1 inhibitor in HTR-8/SVneo cells. Representative images of immunoblot (C) and immunofluorescence (D) show protein expression of Sp1 when the cells were transfected with Sp1 mimic and treated with its inhibitor. (D) Nuclei were stained with DAPI; scale bar: 50 µm. Experiments were performed three times in triplicate (n = 9). Data are presented as mean ± SEM. Paired t-test (mRNA expression of Sp1) and Wilcoxon matched-pairs signed-rank test (protein expression of Sp1) were applied, and p ≤ 0.05 was considered statistically significant. ** denotes p ≤ 0.01; *** denotes p ≤ 0.001. Sp1: specificity protein 1; DAPI: 4′,6-diamidino-2-phenylindole; mRNA: microRNA

Sp1 improves the invasive capacity of HTR-8/SVneo cells. To evaluate the role of Sp1 in HTR-8/SVneo cells, gain- and loss-of-function constructs were optimized (Figure 6).

**Figure 7:**
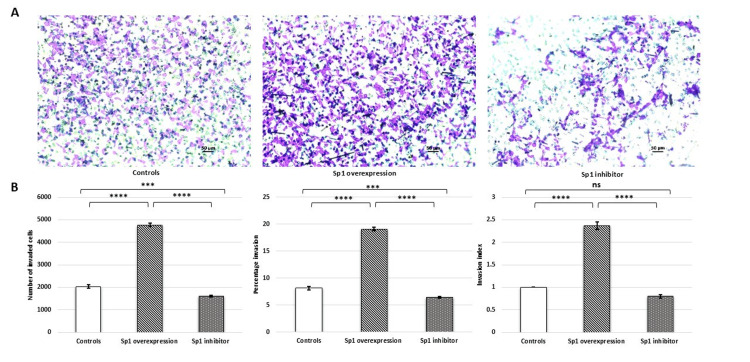
Sp1 mimic increased invasion of HTR-8/SVneo cells whereas its inhibitor decreased the invasion capacity. (A) Representative Matrigel transwell invasion assay images showing invaded cells on the underside of the membrane when no treatment was given (control), and after transfection with Sp1 mimic and treatment with its inhibitor; scale bar: 50 µm. (B) Bar diagrams represent the number of invaded cells, percentage invasion, and invasion index among various experimental groups. Experiments were performed three times in triplicate (n = 9). Sixteen fields were used to count cells on each transwell insert. Data are presented as mean ± SEM. One-way ANOVA, with Bonferroni’s post hoc test, was applied, and p ≤ 0.05 was considered statistically significant. *** denotes p ≤ 0.001; **** denotes p ≤ 0.0001; ns denotes not significant. Sp1: specificity protein 1; ANOVA: analysis of variance

The Sp1 overexpression construct induced a significant upregulation of Sp1 mRNA and protein expression. Conversely, treatment with the Sp1 inhibitor (2,4,5-trifluoroaniline) significantly downregulated Sp1 mRNA and protein expression in HTR-8/SVneo cells. Interestingly, the placental cell line transfected with Sp1 displayed a significantly increased invasion capacity (number of invaded cells, percentage invasion, and invasion index), which decreased upon treatment with the Sp1 inhibitor (2,4,5-trifluoroaniline) (Figure 7).

**Figure 8:**
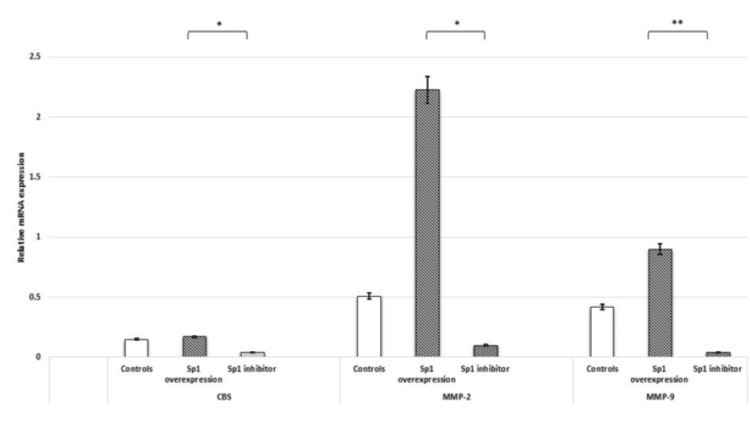
Sp1 upregulates the expression of H2S-synthesizing enzyme (CBS) and MMPs, MMP-2 and MMP-9, in HTR-8/SVneo cells. Bar diagrams represent gene expression of Sp1, CBS, and MMPs 2 and 9 post-Sp1 mimic transfection and treatment with Sp1 inhibitor. Experiments were performed three times in triplicate (n = 9). Data are presented as mean ± SEM. The Wilcoxon matched-pairs signed-rank test was applied, and p ≤ 0.05 was considered statistically significant. * denotes p ≤ 0.05; ** denotes p ≤ 0.01. Sp1: specificity protein 1; CBS: cystathionine β-synthase; H2S: hydrogen sulfide; MMPs: matrix metalloproteinases

Sp1 regulates the expression of H₂S-synthesizing enzymes and MMPs. Overexpression of Sp1 significantly elevated the mRNA expression of CBS, MMP-2, and MMP-9. Treatment of HTR-8/SVneo cells with the Sp1 inhibitor significantly reduced the gene expression of CBS, MMP-2, and MMP-9 (Figure 8).

**Figure 9:**
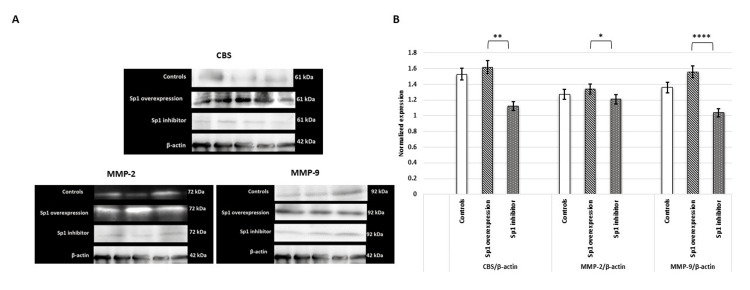
HTR-8/SVneo cells transfected with Sp1 mimic had increased protein expression of Sp1, H2S-producing enzyme (CBS), and MMPs. (A) Representative images of immunoblot showing the protein expression of CBS, MMP-2, and MMP-9 when HTR-8/SVneo cells were transfected with Sp1 mimic and treated with its inhibitor. (B) Bar diagrams represent the normalized values of CBS, MMP-2, and MMP-9 with respect to β-actin (loading control) post-Sp1 mimic transfection and treatment with its inhibitor. Experiments were performed three times in triplicate (n = 9). Data are presented as mean ± SEM. Statistical analysis was done using a paired t-test, and p ≤ 0.05 was considered statistically significant. * denotes p ≤ 0.05; ** denotes p ≤ 0.01; **** denotes p ≤ 0.0001. Sp1: specificity protein 1; CBS: cystathionine β-synthase; H2S: hydrogen sulfide; MMPs: matrix metalloproteinases

**Figure 10:**
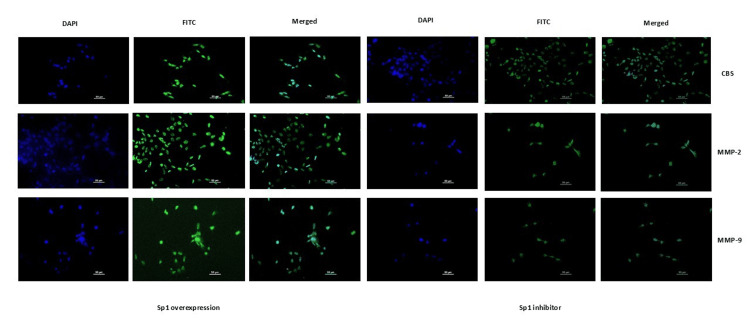
HTR-8/SVneo cells transfected with Sp1 mimic had increased protein expression of Sp1, H2S-producing enzyme (CBS), and MMPs. Representative immunofluorescence images showing localization of Sp1, CBS, MMP-2, and MMP-9 proteins stained with FITC when HTR-8/SVneo cells were transfected with Sp1 mimic and treated with its inhibitor. Nuclei were stained with DAPI; scale bar: 50 µm. Sp1: specificity protein 1; CBS: cystathionine β-synthase; H2S: hydrogen sulfide; MMPs: matrix metalloproteinases; FITC: fluorescein isothiocyanate; DAPI: 4′,6-diamidino-2-phenylindole

The dysregulated expression of transcripts upon overexpression and inhibition of Sp1 was validated at the protein level using immunoblot and immunofluorescence assays. Data demonstrated enhanced expression of CBS, MMP-2, and MMP-9 proteins when the cells were transfected with the Sp1 overexpression construct, but when the cells were treated with 2,4,5-trifluoroaniline, weak expression of CBS, MMP-2, and MMP-9 proteins was observed (Figures 9-10).

**Figure 11:**
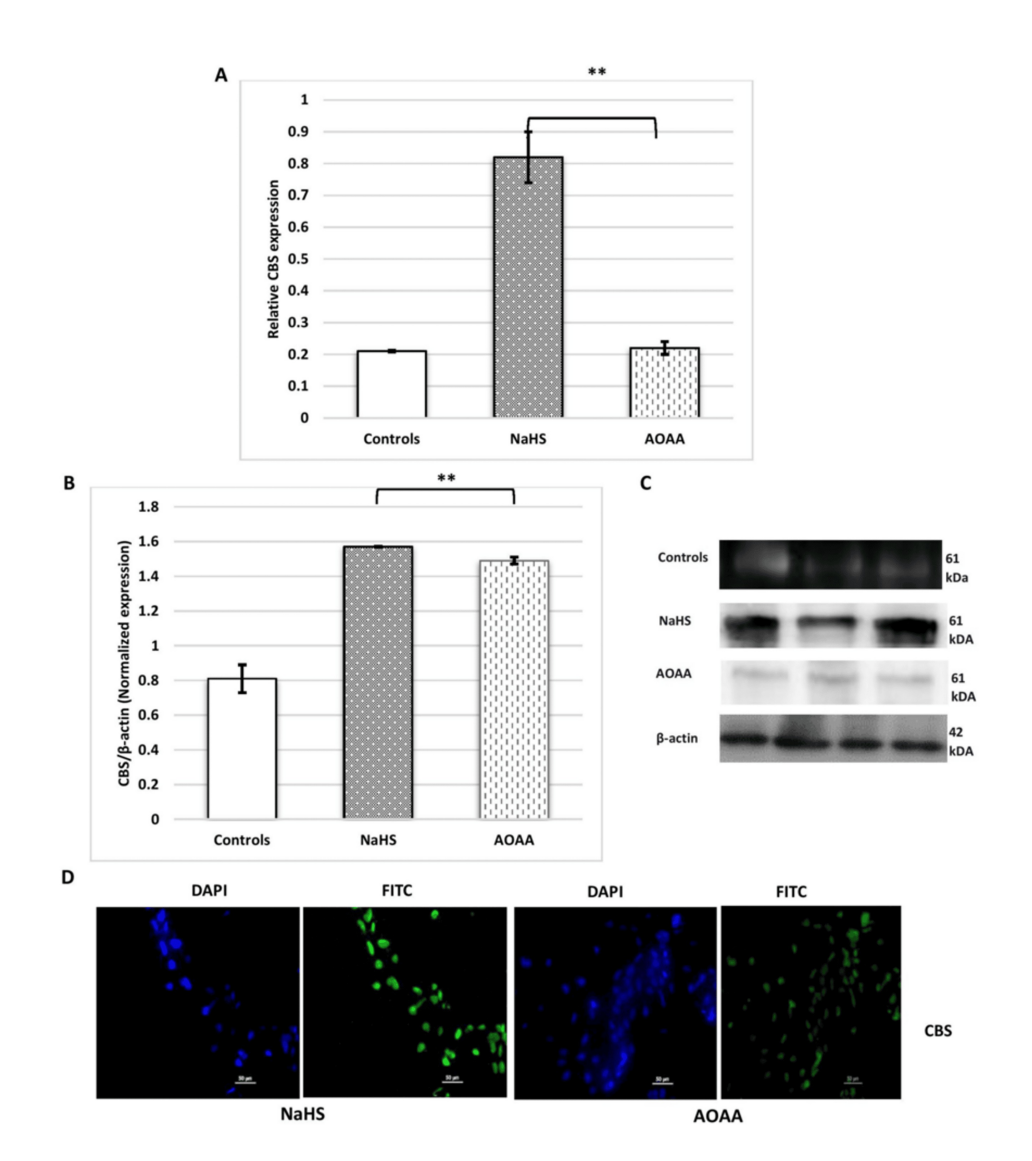
(A, B) Bar diagrams, (C) representative images, and (D) immunofluorescence. Bar diagrams represent mRNA (A) and protein expression (B) of CBS when no treatment was given (controls), and after treatment with NaHS (H2S donor) and AOAA (CBS inhibitor) in HTR-8/SVneo cells. Representative images of immunoblot (C) and immunofluorescence (D) show protein expression of CBS when the cells were treated with NaHS and AOAA. Nuclei were stained with DAPI; scale bar: 50 µm. Experiments were performed three times in triplicate (n = 9). Data are presented as mean ± SEM. Paired t-test was applied, and p ≤ 0.05 was considered statistically significant. ** denotes p ≤ 0.01. mRNA: microRNA; CBS: cystathionine β-synthase; NaHS: sodium hydrogen sulfide; H2S: hydrogen sulfide; AOAA: aminooxy acetic acid; DAPI: 4′,6-diamidino-2-phenylindole

NaHS (H₂S donor) improved the invasion capacity of HTR-8/SVneo cells. Treatment of cells with NaHS (H₂S donor) upregulated the expression of the H₂S-synthesizing enzyme. Significant upregulation of CBS mRNA and protein expression was observed when HTR-8/SVneo cells were treated with NaHS. In contrast, a considerable decrease in CBS expression at both the transcript and protein levels was observed when the cells were treated with AOAA, an inhibitor of CBS (Figure 11).

**Figure 12:**
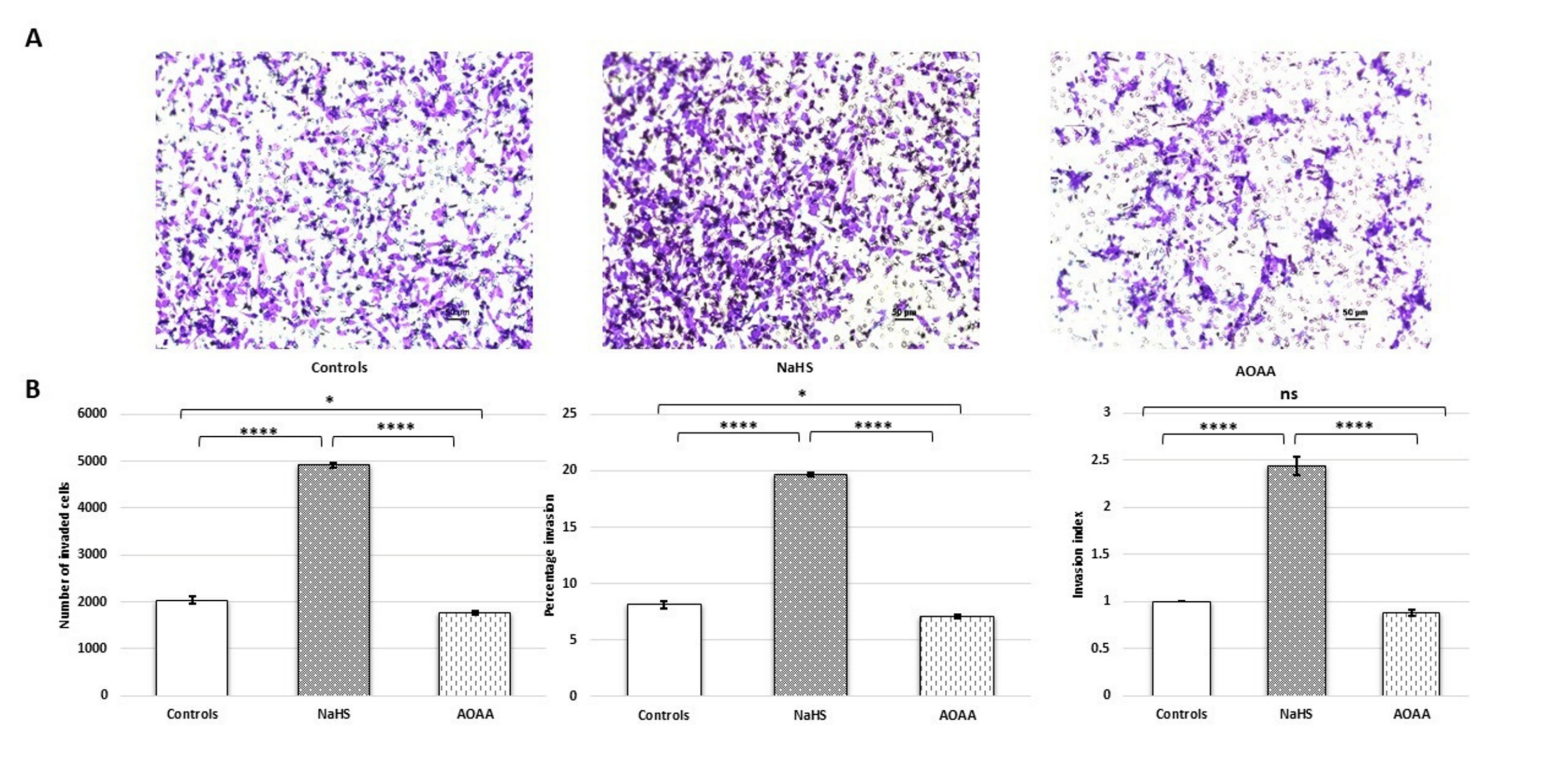
Hydrogen sulphide donor (NaHS) increased the invasive capacity of HTR-8/SVneo cells. (A) Representative Matrigel transwell invasion assay images showing invaded cells on the underside of the membrane when no treatment was given (control) and after treatment with NaHS (H2S donor) and AOAA (CBS inhibitor). Scale bar: 50 µm. (B) Bar diagrams represent the number of invaded cells, percentage invasion, and invasion index among various experimental groups. Experiments were performed three times in triplicate (n = 9). Sixteen fields were used to count cells on each transwell insert. Data are presented as mean ± SEM. One-way ANOVA with Bonferroni’s post hoc test was applied, and p ≤ 0.05 was considered statistically significant. * denotes p ≤ 0.05; **** denotes p ≤ 0.0001; ns denotes not significant. CBS: cystathionine β-synthase; NaHS: sodium hydrogen sulfide; H2S: hydrogen sulfide; AOAA: aminooxy acetic acid

The Transwell invasion assays observed a significant improvement in the invasion capacity (increase in the number of invaded cells, percentage invasion, and invasion index) of HTR-8/SVneo cells following treatment with NaHS. The invasive capacity was observed to be reduced upon treatment with AOAA (Figure 12).

**Figure 13:**
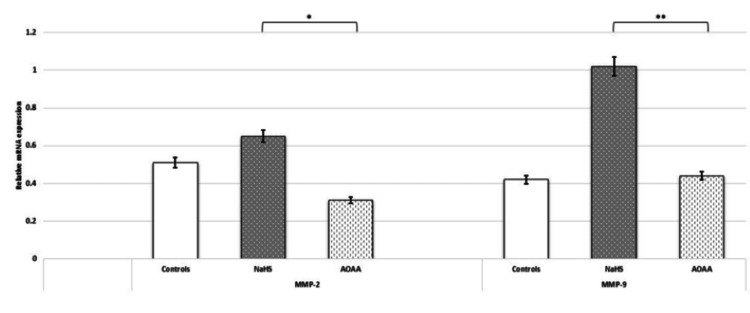
H2S donor (NaHS) upregulated the expression of MMPs in HTR-8/SVneo cells. Bar diagrams represent gene expression of MMP-2 and MMP-9 post NaHS (H2S donor/CBS mimic) and AOAA (CBS inhibitor) treatments. Experiments were performed three times in triplicate (n = 9). Data are presented as mean ± SEM. The Wilcoxon matched-pairs signed rank test was applied, and p ≤ 0.05 was considered statistically significant. * denotes p ≤ 0.05; ** denotes p ≤ 0.01. CBS: cystathionine β-synthase; NaHS: sodium hydrogen sulfide; H2S: hydrogen sulfide; AOAA: aminooxy acetic acid; MMPs: matrix metalloproteinases

NaHS (H₂S donor) upregulated the expression of MMPs. Regulation of MMP-2 and MMP-9 by H₂S via CBS was investigated, and the mRNA expression of these genes was significantly upregulated upon treatment of HTR-8/SVneo cells with NaHS. These results corroborated the significant decrease in the gene expression of MMP-2 and MMP-9 when the cells were treated with the CBS inhibitor AOAA (Figure 13).

**Figure 14:**
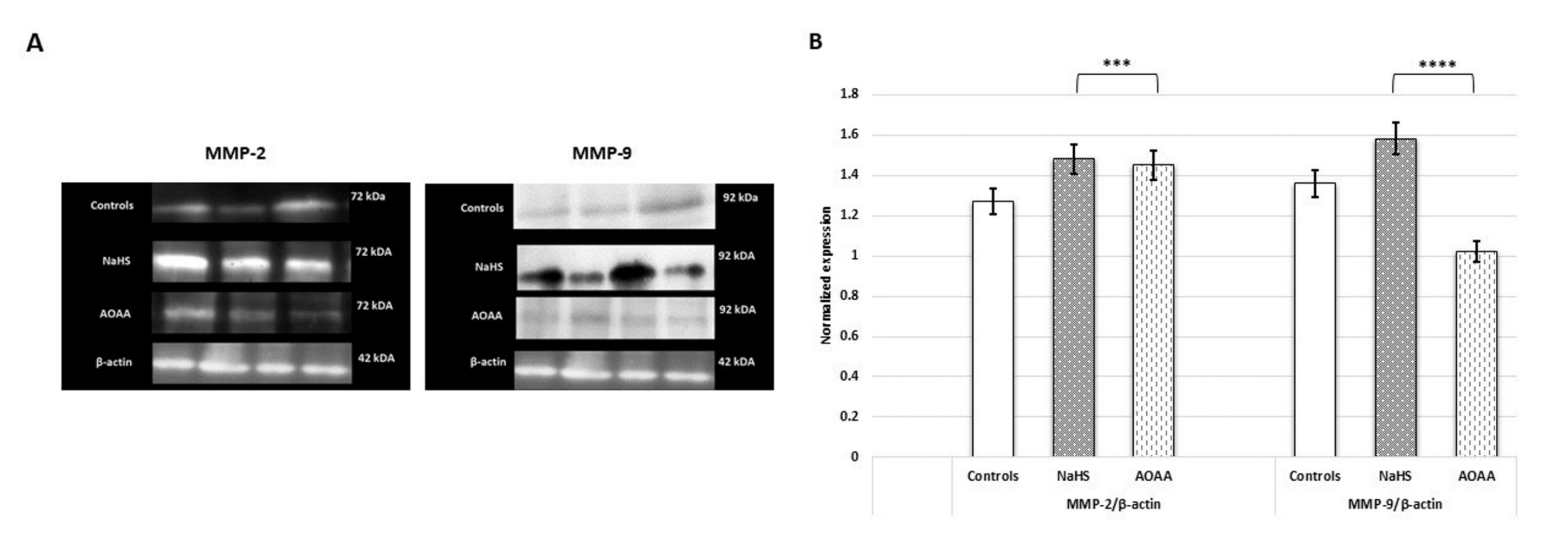
H2S donor (NaHS) increased the expression of MMPs enzymes. (A) Representative immunoblot images showing the protein expression of MMP-2 and MMP-9 when HTR-8/SVneo cells were treated with NaHS (H2S donor) and AOAA (CBS inhibitor). (B) Bar diagrams represent the normalized values of MMP-2 and MMP-9 with respect to β-actin (loading control) post NaHS and AOAA treatments. Experiments were performed three times in triplicate (n = 9). Data are presented as mean ± SEM. Statistical analysis was done using a paired t-test, and p ≤ 0.05 was considered statistically significant. *** denotes p ≤ 0.001; **** denotes p ≤ 0.0001. CBS: cystathionine β-synthase; NaHS: sodium hydrogen sulfide; H2S: hydrogen sulfide; AOAA: aminooxy acetic acid; MMPs: matrix metalloproteinases

**Figure 15:**
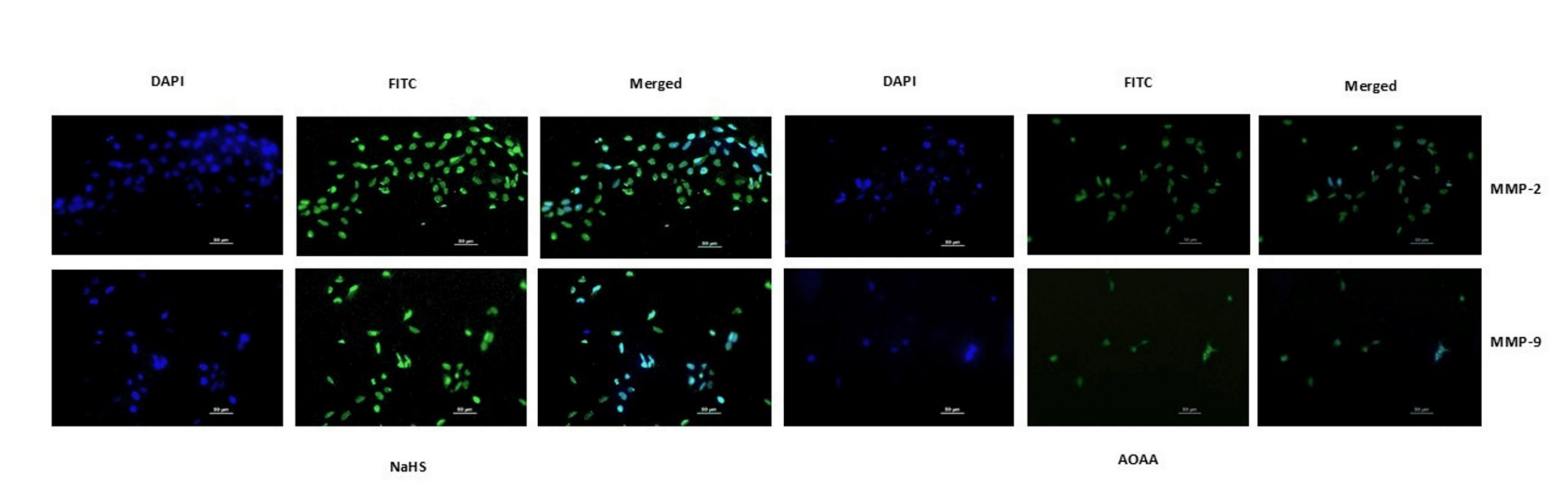
H2S donor (NaHS) up regulated whereas AOAA down regulated the expression of MMP-2 and MMP-9 proteins. Representative immunofluorescence images showing localization of CBS, MMP-2 and MMP-9 proteins stained with FITC when HTR-8/SVneo cells were treated with NaHS (H2S donor/CBS mimic) and AOAA (CBS inhibitor). Nuclei were stained by DAPI, scale bar: 50 µm. CBS: cystathionine β-synthase; NaHS: sodium hydrogen sulfide; H2S: hydrogen sulfide; AOAA: aminooxy acetic acid; MMPs: matrix metalloproteinases; DAPI: 4′,6-diamidino-2-phenylindole; FITC: fluorescein isothiocyanate

Immunoblotting and immunostaining revealed strong signals of MMP-2 and MMP-9 proteins upon NaHS treatment, compared to the weakened protein signals observed when treated with AOAA (Figures 14-15).

## Discussion

miRNAs identified in human placental tissues have been recognized as crucial regulators of placental development [2-8]. Anomalies in the expression of miRNAs in trophoblast cells from women with compromised pregnancies have been reported [3,4,12,13]. Along with miRNAs, the role of gasotransmitters, like NO and H₂S, in placentation has also been explored recently, albeit previous studies from our group have demonstrated that H₂S promotes trophoblast cell migration and rescues them from hypoxia-reperfusion injury [27].

With the hypothesis that enzymes involved in H₂S synthesis, which influence trophoblast cell invasion, may potentially be regulated by miRNAs, we delved into these upstream mechanisms controlling the enzymes. We demonstrate that trophoblast cells (HTR-8/SVneo) transfected with miR-22 significantly reduced their invasion capacity, accompanied by downregulation of MMP-2 and MMP-9. Furthermore, these cellular alterations induced by miR-22 were associated with the downregulation of CBS, an enzyme responsible for H₂S synthesis.

Elevated expression of miR-22 has been reported in the placentae of pregnancies complicated by PE, and especially in EOPE [14]. Published literature in the field of cardiovascular diseases reports inhibition of Sp1, a transcription factor for CSE, by miR-22 [28]. These reports validate our hypothesis whereby, in PE, the underlying cause of reduced expression of MMP-2 and MMP-9 in pregnancy complications associated with placental functions could be the regulation of Sp1 by miR-22 in the trophoblasts. MMP-2 and MMP-9 are key players in regulating trophoblast cell invasion by facilitating the degradation of the extracellular matrix, aiding in the invasion of spiral arteries, and transforming these vessels into low-resistance, high-capacitance vessels [25]. The reduced expression of MMP-2 and MMP-9 in preeclamptic and intrauterine growth restriction (IUGR) placentae has been extensively documented [26,29]. Worldwide studies have identified multiple miRNAs targeting MMP-2 and MMP-9, potentially contributing to the pathogenesis of PE and IUGR [30]. In colon cancer cells, miR-22 transfection has been reported to significantly reduce the expression of MMP-2 and MMP-9 [10].

To our knowledge, this is the first report to demonstrate the impact of miR-22 on the mRNA and protein expression of MMP-2 and MMP-9 in human placental HTR-8/SVneo cells. miR-22 has been identified as a tumor-suppressive factor, downregulating oncogenic target genes in various cancers [10]. Therefore, we further identified miR-22 targets in HTR-8/SVneo cells and studied the underlying mechanism of miR-22 regulation of MMP-2 and MMP-9. This suggests that the cellular remodeling induced by H₂S is regulated by the expression of miR-22.

In order to study the underlying mechanism of miR-22 regulation of MMP-2 and MMP-9 in placental cells, a miR-22-3p mimic was transfected, which caused a significant downregulation in the expression of Sp1.

Interestingly, transfection of Sp1 mimic/Sp1 overexpression construct demonstrated significant upregulation of MMP-2 and MMP-9, with increased invasive capacity. Sp1 has been identified as a direct target of miR-22 in gastric tumors, with an inverse linear correlation between miR-22 expression and Sp1 mRNA [15].

Sp1 overexpression in trophoblast cells led to significant upregulation of CBS's mRNA and protein expression. Sp1 has been reported to play a major role in the regulation of CBS [17]. CBS, in turn, regulates homocysteine metabolism and contributes to H₂S biosynthesis, playing diverse roles in regulating cellular energetics, redox status, DNA methylation, and protein modification. Studies on Sp1-deficient fibroblast cells, upon transfection with an Sp1 expression construct, have reported high levels of CBS expression [17].

In this study, to our knowledge, we have demonstrated for the first time an association between miR-22 and H₂S in HTR-8/SVneo cells. Transfection with a miR-22-3p mimic led to a significant decrease in CBS’s mRNA and protein expression. In contrast, transfection with a miR-22-3p inhibitor resulted in a substantial increase in CBS mRNA and protein expression. Additionally, upregulation of MMP-2 and MMP-9 was observed upon transfection with a miR-22-3p inhibitor, thus validating that changes in cellular activities were linked to the miR-22-3p-CBS-MMP-2 and MMP-9 axis through regulation of Sp1 by miR-22-3p.

Similarly, treatment of HTR-8/SVneo cells with exogenous NaHS (H₂S donor) notably upregulated the expression of MMP-2 and MMP-9, along with a significant increase in invasion capacity. Our findings are consistent with a study reported on human bladder cancer cells, where treatment with varying concentrations of NaHS (exogenous H₂S donor) led to a significant increase in the expression levels of MMP-2 and MMP-9, along with enhanced invasion capacity [23].

Our investigation identified a miR-22/Sp1/CBS axis that regulates MMP-2 and MMP-9 expression. The study further demonstrates that miR-22 significantly repressed the invasion of trophoblast cells in vitro by downregulating MMP-2 and MMP-9. MiR-22 appears to achieve this repression indirectly by binding to the three-prime untranslated region (3′-UTR) of Sp1 [15].

Although Sp1 is also known to bind to the MMP-2 promoter and enhance its expression, the direct effect of Sp1 on MMP-2, MMP-9, or via CBS needs further elucidation [17]. Nevertheless, to our knowledge, we present the first evidence of the pivotal role of miR-22 in modulating MMP-2 and MMP-9 expression in trophoblast cell invasion.

This study unveils the significant role of miR-22 in influencing the invasive potential of placental cells - a critical and essential process during placental development, disruptions in which potentially lead to pregnancy-related complications and adverse pregnancy outcomes. 

The dysregulated miR-22/Sp1/CBS/H₂S/MMPs 2,9 axis may be regarded as one of several putative factors present in patients’ sera, which not only contributes to various stresses but also reduces angiogenesis, migration, and invasion, thereby playing an important role in maternal signs and symptoms of PE. There is a need to identify the various intracellular signalling pathways that could be triggered by H₂S biosynthesis in trophoblast cells using an in vitro cell culture approach, which will help in identifying druggable targets. The candidate genes thus identified can be verified using placental cell lines or primary trophoblast cells to better understand their role in the disease. Additionally, validation of selected genes in human placental tissue or circulating maternal blood will provide intriguing insights into the role of H₂S in the etiology of this enigmatic disease with multiple phenotypes.

The insights gained from our research contribute to a deeper understanding of the molecular mechanisms through which CBS and MMPs promote trophoblast cell invasion. Moreover, these findings may pave the way for developing novel therapeutic targets and for future therapies of placenta-related pregnancy complications, such as PE or IUGR.

## Conclusions

The results from the present study may point towards the role or effect of miR-22 in the invasion of trophoblast cells by regulating the levels of MMP-2 and MMP-9 via the Sp1 and CBS pathway. To our knowledge, the present study, for the first time, provides novel insights into the effects of miR-22, Sp1, and CBS on the invasion capacity of trophoblast cells. The role of the miR-22/Sp1/CBS axis in regulating trophoblast invasion by influencing the levels of MMP-2 and MMP-9 may become foundational knowledge, ultimately contributing to the investigation, clarification, and prevention of pregnancy-related complications such as PE, recurrent pregnancy loss, and fetal growth restriction (FGR), while also providing insights for pharmacological interventions in these conditions.
